# Usability Challenges in Electronic Health Records: Impact on Documentation Burden and Clinical Workflow: A Scoping Review

**DOI:** 10.1111/jep.70189

**Published:** 2025-06-29

**Authors:** Olufisayo Olakotan, Ray Samuriwo, Hadiza Ismaila, Samuel Atiku

**Affiliations:** ^1^ Department of Neonatology, Women and Children's Directorate University Hospitals Leicester NHS Trust Leicester UK; ^2^ School of Health and Social Care Edinburgh Napier University, Sighthill Campus, Sighthill Court Edinburgh Scotland UK; ^3^ Centre for Digital Innovations in Health and Social Care (CDIHSC), Faculty of Health Studies University of Bradford; Bradford England UK; ^4^ Digital Technology and Innovation University of Staffordshire UK; ^5^ Research Services Aston University Birmingham UK

**Keywords:** clinical workflow, data entry, documentation burden, electronic health records, interface usability

## Abstract

**Background:**

The adoption of Electronic Health Records (EHRs) has become integral to today's healthcare by supporting preventive care; however, it often imposes significant documentation burdens that disrupt workflows. These challenges may stem from usability issues driven by system or interface design flaws that result in the misalignment of EHR with clinical workflows, increasing clinicians' cognitive load. This study aims to identify and analyze the usability issues contributing to documentation burdens and subsequently lead to workflow disruptions.

**Methods:**

The scoping review employed the methodology developed by Levac. Three databases, namely PubMed, Scopus, and Ovid MEDLINE, were searched to identify relevant studies published in English between 2007 and 2024. Handsearching of key journals was also conducted to ensure comprehensive coverage of the literature. All findings were reported according to PRISMA guidelines for scoping reviews.

**Results:**

Of 2387 identified records, only 28 studies met the inclusion criteria, employing qualitative, mixed methods as well as time‐motion studies. The studies noted that clinicians frequently experienced significant workflow disruptions caused by poorly designed interfaces, which led to task‐switching, excessive and prolonged screen navigation, and fragmented critical information across EHR. These challenges often necessitated workarounds, such as duplicating documentation and using external tools, further increasing the risk of data entry errors and prolonging documentation times.

**Conclusion:**

Our study findings highlight the critical need for improved EHR design that minimises workflow disruptions associated with documentation burden. Addressing these challenges requires human factors approach that streamlines information retrieval, optimizes interface usability, and eliminates unnecessary task complexity.

## 1 Introduction

Electronic Health Records (EHRs) have become foundational to 21st‐century healthcare, with their widespread adoption driven by efforts to improve care coordination, enhance data accessibility, and enable population‐level analytics [1, 2]. Policy initiatives such as the U.S. Health Information Technology for Economic and Clinical Health (HITECH) Act significantly accelerated this uptake, resulting in near‐universal adoption across U.S. healthcare institutions [3, 4]. As of 2021, 96% of nonfederal acute‐care hospitals and 78% of office‐based physicians in the U.S. use an EHR, making these systems integral to routine clinical practice [5].

However, alongside these advancements, EHRs have introduced considerable challenges, foremost among them is documentation burden [6]. Clinicians now spend an estimated one‐third to one‐half of their workday interacting with EHR systems, translating to over $140 billion in lost care capacity annually [7, 8]. The most time‐intensive activities include chart review, order entry, and inbox management [9]. Despite their ubiquity, EHRs lack standardized and robust metrics for documentation burden, and clinicians consistently report that documentation disrupts their workflow, limits time with patients, and contributes to professional dissatisfaction [10, 11, 12, 13].

Research attributes this burden not only to the volume of documentation required but also to poor system usability, limited interoperability, and misaligned workflows [10, 11, 12, 13]. For example, physicians in the U.S. have rated their EHRs with a median System Usability Scale (SUS) score of just 45.9/100, placing them in the bottom 9% of all software systems [14, 15]. Each one‐point drop in SUS has been associated with a 3% increase in burnout risk [14, 15]. Empirical studies show that poor interfaces, deep menu hierarchies, and poor data searchability significantly extend task completion times and elevate cognitive load [16, 17]. Additional usability issues, such as repetitive data entry, lack of automation, and weak user guidance have been linked to increased note length, duplicated content, and documentation errors [18, 19].

This scoping review aims to address these gaps by systematically mapping the literature on EHR usability challenges that contribute to documentation burden and clinical workflow disruption. By identifying key themes, patterns, and gaps, this review seeks to inform future EHR design, policy, and implementation strategies that better align with clinician needs and patient care priorities.

## 2 Methods

This scoping review was conducted following the methodological framework originally proposed by Arksey and O'Malley (2005) [20] and further expanded by Levac et al. (2010) [21]. This approach facilitates the exploration of the extent, range, and nature of research activity, aiming to identify all relevant literature in the field. The conduct and reporting of this scoping review align with the PRISMA Extension for Scoping Reviews (PRISMA‐ScR) checklist.

**The research question:** What are the usability issues in EHR systems that contribute to documentation burden and clinical workflow disruption? Within the context of our work, we define usability issues as inefficiencies in EHR systems stemming from either system or interface design flaws [11]. These inefficiencies contribute to documentation burden, increase the time and effort required for clinicians to complete documentation tasks, and disrupt the natural flow of work.

**Search strategy:** Three electronic databases PubMed, Scopus, and Ovid MEDLINE were searched to identify relevant studies published between 2007 and 2024. These databases were selected to ensure comprehensive coverage of the literature, as they index a broad range of peer‐reviewed studies across multiple disciplines. The initial searches were conducted in May 2024 and updated in August 2024 using a combination of keywords, MeSH terms, and database‐specific search strings. Additionally, the reference lists of relevant studies were manually reviewed to identify further studies.

**Study selection:** This review included empirical studies that used quantitative, qualitative, or mixed‐method approaches (e.g., time‐motion studies, cognitive load assessments, interviews, focus groups) to explore EHR usability, documentation burden, and workflow inefficiencies in clinical settings. Eligible studies addressed clinician‐facing challenges related to system and interface design flaws, such as task‐switching, redundant data capture, workarounds, system integration issues, and data entry difficulties (e.g., manual input, auto‐population errors, navigation complexities) (Table 1). Research focusing on the impact of these challenges on documentation processes was also included, even if documentation burden was not explicitly mentioned.

**Table 1:** Inclusion and exclusion criteria for study selection.

PCC Component	Inclusion	Exclusion
Population	Studies investigating healthcare professionals' use of EHR systems in clinical practice, including physicians, nurses, and allied health professionals.	Studies focusing exclusively on administrative staff, patients, or general IT adoption unrelated to clinical practice.
Concept	– Studies addressing interface design issues (e.g., navigation challenges, template inefficiencies, alerts, or data entry processes) and their impact on workflow disruptions or misalignment with clinical practices. – Studies on redundant data capture, double documentation, or inefficiencies in recording patient information, including the associated time, effort, and cognitive load. – Studies exploring EHR integration with other clinical systems (e.g., laboratory systems, imaging tools) and challenges in accessing or sharing patient information. – Studies examining workarounds (e.g., external note‐taking tools, copying and pasting data) to address usability or workflow challenges. – Studies evaluating tools/technologies (e.g., speech recognition, AI‐based systems) to mitigate usability challenges or reduce documentation burden.	– Studies not focusing on documentation burden or data entry as primary topics, or failing to explicitly mention or measure these challenges in clinical documentation. – Studies addressing general EHR usability without specific reference to documentation tasks or clinical workflows (e.g., billing, administrative tasks). – Studies focusing on outcomes like burnout, collaboration, or patient outcomes without linking these to documentation burden. – Studies focusing on LLM or AI tools to alleviate documentation burden without mentioning usability or workflow issues. – Studies on EHR medication alerts without mentioning their contribution to documentation burden.
Context	Studies conducted in clinical settings (e.g., hospitals, outpatient clinics, primary care) using methodologies like time‐motion analysis, qualitative, quantitative, or mixed‐method research.	Studies conducted in nonclinical settings (e.g., education or purely IT‐related environments) or that are systematic reviews, editorials, or opinion pieces without unique insights into EHR usability.

Excluded were studies focusing on non‐EHR systems, nonclinical settings, outdated technologies, or general IT adoption without specific reference to EHR usability, documentation burden, or workflow issues. Articles lacking primary empirical data, such as systematic reviews, opinion pieces, and editorials, or those focusing solely on outcomes like burnout, patient satisfaction, or medication alerts, were excluded. Additionally, research on AI or LLM tools for reducing documentation burden was excluded unless it explicitly addressed usability and workflow integration challenges.

All articles retrieved from the selected databases were uploaded to Rayyan, a systematic review software designed to assist with de‐duplication, filtering, and selection of publications for inclusion in systematic and scoping reviews. One author (OO) independently screened the article titles, while two reviewers (OO, OS) screened the abstracts. Conflicting decisions were resolved through discussion between the two reviewers. In cases where consensus could not be reached, an independent third reviewer was consulted to make the final decision. Subsequently, two reviewers (OO, OS) screened the full‐text articles, resolving any disagreements through discussion. For the final selection of full‐text articles, the two reviewers (OO, OS) met in person to reach a consensus, with (HS) available if needed.

**Charting the data:** A standardized data extraction form was used to collect information on authors, year, objectives, methodology, study setting, location, key findings, concise summaries, and research gaps from selected articles. First‐order coding, conducted by (OO and OS), assigned descriptive labels to key patterns or problems in the data without over‐interpretation. This was followed by second‐order coding, allowing (OO and OS) to refine initial codes and identify patterns across studies. Once finalized, these codes were synthesized into broader themes representing recurring issues across multiple studies.

Finally, the quality of all selected articles was assessed using the Mixed Methods Appraisal Tool (MMAT), which is designed for appraising systematic mixed studies reviews, including qualitative, quantitative, and mixed methods designs. The quality of each paper was assessed using a yes‐or‐no checklist, and an overall score was calculated as a percentage. Papers scoring ≥75% were classified as high quality, while those scoring between 60% and 74% were considered acceptable. Results from the quality assessment show that 20 papers are of high quality, while the remaining five papers have acceptable quality, meaning they are still considered sufficiently rigorous and reliable for inclusion.

**Data analysis and synthesis:** A descriptive summary of quantitative (e.g., time‐motion results) and qualitative (e.g., clinician feedback) data was integrated to fully understand documentation and usability issues by comparing findings from various studies. A narrative synthesis was used to group findings under broad themes, patterns, and trends across different studies or settings, aligning with our study objectives and supporting the synthesis of findings where needed.

## 3 Results

**Figure 1:**
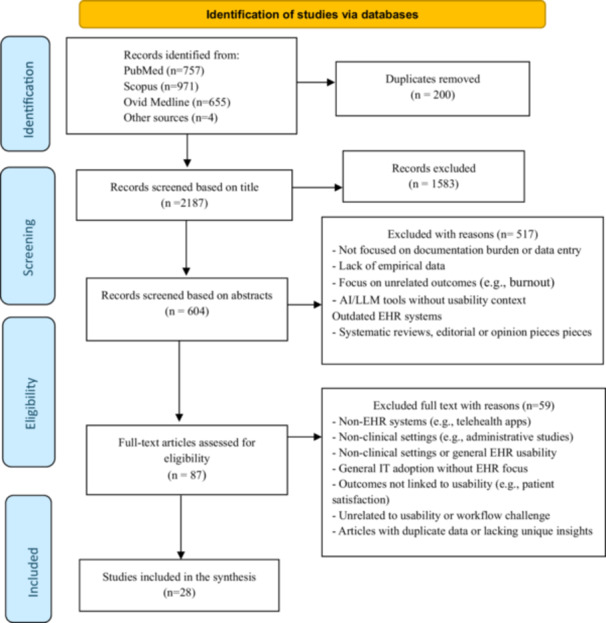
PRISMA diagram outlining study selection from the data sources included in literature search.

The bibliographic databases returned 2387 publications. The numbers of articles retrieved from each database are as follows: Scopus (*n* = 971), PubMed (*n* = 757), Ovid Medline (*n* = 655), Other sources (*n* = 4). Duplicates were removed (*n* = 200). Then, 2187 titles and abstracts were initially screened based on their title and abstracts, and 1538 were excluded due to the exclusion reasons stated in Figure 1. Upon full‐text screening records, only 87 articles were accessed, and 59 papers were further excluded because of their irrelevance to the research question.

### 3.1 Overview of Study Design

The summaries of the 28 articles included in the review are shown in (appendix A). Different methodologies were employed to explore usability challenges associated with documentation burden in EHR systems. Think‐aloud protocols and scenario‐based usability testing were used in two studies to identify EHR usability challenges [22, 23]. Mixed‐methods approaches, integrating qualitative and quantitative data for a comprehensive analysis, were employed in six studies [24, 25, 26, 27, 28, 29]. Qualitative methods, such as interviews and observations, were used in three studies to explore provider experiences and document workarounds [30, 31, 32].

Similarly, five studies relied on cross‐sectional surveys to gather data on usability perceptions, burnout, and documentation practices [33, 34, 35, 36, 37]. Time‐motion methodologies were employed in three studies to observe workflows and evaluate EHR‐related tasks [38, 39, 40]. Retrospective analyses were used in three studies to identify documentation patterns and workflow inefficiencies [41, 42, 43]. Additionally, two studies focused on iterative design and implementation approaches to enhance EHR usability [44, 45], while two others used exploratory methods and expert interviews to understand barriers in EHR systems [46, 47]. One used Heuristic evaluation identified EHR design flaws influencing usability and workload [48]. Another used Before‐and‐after study involving video analysis of consultations [49].

The studies were conducted in various healthcare settings, including Academic Medical Centres & University Hospitals (*n* = 14), General & Specialty Hospitals (*n* = 4), Primary Care & Outpatient Clinics (*n* = 4), Ambulatory & Urgent Care Settings (*n* = 1), Specialised Healthcare Institutions (*n* = 2), Residency & Training Settings (*n* = 2), Pharmacy‐Based Settings (*n* = 1), and Regional care Settings (*n* = 2). The studies were conducted in the following countries: USA (n = 19), Canada (*n* = 2), Japan (*n* = 1), Germany (*n* = 1), Sweden (*n* = 1), and the Netherlands (*n* = 1). We have provided Table 2, which outlines the key issues for each theme drawn from our study, alongside the desired EHR features and functions from a human factor perspective.

**Table 2:** Key usability issues and desired key features/functions.

Themes	Key issues	Desired key features & functions
Task‐switching and workflow fragmentation	– Frequent task switching (1.4 times/min), leading to fragmented attention and disrupted clinical workflow. – Repeated interruptions to perform data entry, order entry, and data viewing. – Documentation duplication (e.g. paper notes later transcribed into EHR).	– Use user‐centered interface designs that consolidate tasks (e.g. inline order entry, embedded data viewing), minimising screen changes. – Implement interruption management strategies (e.g. queued nonurgent notifications) so clinicians can complete core tasks without fragmenting attention. – Provide a split‐screen or multi‐tab layout that allows clinicians to keep key information visible while performing related tasks.
Excessive cognitive demands and information complexity	– High number of keystrokes and data input demands increase clinicians' cognitive load. – Complex interface designs and poorly organized information. – Difficulty differentiating critical from noncritical results.	– Present data in logical groupings, use progressive disclosure so that complex details appear only when needed. – Flag and highlight critical results to help clinicians focus on the most urgent items. – Employ structured data fields with autofill or predictive text where possible to cut down on keystrokes and reduce mental overhead. – Provide robust, intuitive filtering or search tools for quick data retrieval.
EHR‐clinical workflow misalignment and inefficient workarounds	– EHR functionalities not aligned with real‐world clinical processes. – Need for copying/pasting information from other sources. – Excessive alerts and pop‐ups disrupt workflow.	– Conduct task analyses and employ iterative design based on clinicians' actual workflows, so the EHR supports rather than disrupts them. – Design integrated modules (e.g. embedded notetaking, integrated scheduling) that eliminate the need for external tools. – Use context‐aware alerts that only surface when clinically relevant, reducing alert fatigue. – Allow each clinician to configure default views, shortcuts, and reminder thresholds to suit individual practice patterns.
Navigation challenges disrupt workflows and lead to errors	– Nonintuitive navigation paths requiring excessive clicking. – Frequent toggling between multiple screens to locate information. – Large open‐ended text areas prone to error and inconsistency.	– Apply consistent menu structures, button placements, and terminology, so clinicians do not waste cognitive resources re‐orienting. – Introduce consolidated views and short, direct paths to commonly accessed functions (e.g. quick links, logical grouping of menu items). – Provide structured templates with clear fields, minimising open‐ended text to reduce data variability and errors. – Allow users to navigate directly to relevant tasks (e.g. from lab results to ordering follow‐up tests) without returning to a main menu.
Fragmented information hinders retrieval, increasing documentation time and errors	– Data scattered across multiple systems (lab, radiology, separate modules). – Clinicians resort to paper lists or verbal updates. – Chronological sorting of notes complicates information retrieval	– Integrate disparate data streams into one cohesive platform or ‘dashboard’ to avoid multiple logins and data silos. – Permit tagging or grouping of notes by problem, specialty, or date to enable quick identification of relevant documents. – Deliver instant updates from labs or radiology into the EHR with prompts that tie directly to a patient's record. – Employ live voice recognition or mobile interfaces to capture data in real time, reducing after‐the‐fact fragmentation.

### 3.2 Task‐Switching and Workflow Fragmentation

Clinicians averaged 1.4 task switches per minute, with rates of 1.5 switches per minute in the ACU, 1.4 in the ED, and 1.3 in the ICU, higher than the 0.9 switches per minute in the ambulatory clinic [50]. Nearly all these switches related to specific tasks, with data viewing accounting for 20.5%, data entry for 7.0%, and order entry for 3.9%, making up nearly half of all task switches [50]. A study found that clinicians frequently switched tasks, with three main screen transition patterns accounting for 19 out of 66 patients (29%), seven additional patterns for 15 patients (23%), and entirely unique patterns for the remaining 32 patients, indicating inefficiencies in information‐gathering [42]. Conversely, Danak et al. (2019) found that scribes had little impact on encounter duration, though scribed encounters were slightly shorter [27]. Physicians spent less time looking at the computer when scribes were present, suggesting that scribes help reduce task‐switching and allow for greater focus on patient interactions. Another study reported a reported a 27% reduction in total EHR time for initial outpatient consultations (IOC), indicating that e‐pathway implementation reduced the time spent on documentation [49]. This improvement made it easier for healthcare professionals to transition seamlessly between direct patient care tasks and EHR‐related activities, ultimately enhancing workflow efficiency.

A time‐motion observation of healthcare professionals in a large academic medical centre reported that workflows are naturally fragmented, as healthcare professionals often need to pivot between patient care tasks [38]. These tasks are frequently interrupted or modified. Additionally, documentation duplication, in which information is recorded on paper at the bedside and later entered into the EHR, or patient information is documented in multiple formats, compounds the documentation burden and highlights the fragmented nature of EHR workflows [30, 39]. This practice caused delays in making information accessible to other clinicians, which negatively impacted communication and disrupted continuity of care, potentially leading to delayed decision‐making and compromised patient outcomes [28].

### 3.3 Excessive Cognitive Demands and Information Complexity

Clinicians face high information input demands due to a 24% increase in physical keystrokes and an additional 102.49 automated keystrokes per initial outpatient consultation, which contributes to cognitive load [48]. They also experienced a doubling of workload after EHR adoption, with no substantial reduction, resulting in a persistently high cognitive burden [37, 40]. This was attributed to the interface complexity of EHR systems and the shift toward real‐time documentation during patient encounters. This cognitive demands imposed by EHR systems led to difficulty in sorting, sifting, and locating relevant documents, as notes are stored by title and arranged chronologically, requiring clinicians to sift through multiple physical therapy notes to access the latest primary care visit [31]. ICU nurses confronted redundant vitals fields and cramped text boxes, reporting “information overload” in interviews; log files confirmed that completing flowsheets with duplicated data entries added 11.6 min per 12‐h shift [29]. Also, the complexity and volume of messages in the EHR, along with the inability to distinguish certain abnormal results from normal ones in the inbox, increase documentation burden and add cognitive strain [47]. A similar study reports that heavily edited dictated notes, including both short and continuation edits, resulted in cognitive strain and increased frustration for clinicians [45]. Short edits accounted for 81% of the edit spans and were a significant source of frustration, as locating these smaller edits required time and attention to detail [43].

### 3.4 EHR‐Clinical Workflow Misalignment and Inefficient Documentation Workarounds

Clinicians reported that EHR‐workflow misalignment led to communication challenges, made patient record review and documentation burdensome, which often extended their workdays by an average of 90 min [25]. In one study, 52% of psychiatrists still listed medication reconciliation and prescription workflows as pain points, and 39% resorted to off‐system notes, evidence of persistent misalignment despite usability interventions [33]. Difficulty finding necessary health information exchange (HIE) tools within the existing workflow led to inefficiencies, with clinicians spending additional time searching for information and deviating from their standard workflow [24]. Additionally, diagnostics, such as lab results that were not seamlessly integrated into the workflow, led to potential delays in reviewing critical patient information, increased the data entry burden, and required physicians to manually check for new results [39]. This EHR‐workflow misalignment led to common workarounds such as inserting additional data, copying and pasting information from previous notes to ensure completeness and accuracy, but this method introduced risks of redundancy and error [25, 43]. Another study reported that clinicians used workarounds to mitigate usability issues, such as composing notes in external applications (e.g., Microsoft Word) before transferring them into the EHR or managing appointments through Outlook instead of directly within the EHR [37]. Clinicians stated that the EHR interface should be designed to better align with familiar elements of their workflow, such as using ‘tabs’ for easier navigation, as they felt the current EHR design complicated the transition between tasks [24, 39]. They stated that documentation was more efficient when these tasks aligned naturally with their personal workflows [20, 23]. Emphasizing further improvements in the length, functionality, and user‐friendliness of reminders, along with better integration within the EHR, can improves workflow alignment by making documentation more seamless and less burdensome [20, 23].

### 3.5 Navigation Challenges in EHR Design Disrupt Workflows and Lead to Documentation Errors

The EHR is frequently described as nonintuitive, requiring excessive navigation through numerous pages and clicks, which forces clinicians to undertake additional administrative tasks not directly related to patient care, contributing to documentation burden [25]. Deep navigator hierarchies and nonintuitive menu labels doubled the clicks required to reach infusion‐pump documentation; wrong‐field data entry occurred in 17% of observed tasks [29]. Clinicians also found it challenging to navigate certain applications, such as the Clinical Data Search, and experienced issues with logging out, leading to excessive clicks needed to access or document data [24]. A study analyzing the inpatient EHR clinical notes documentation interface reported lower satisfaction with the EHR, with a mean SUS score of 60.8, indicating ‘marginal usability,’ compared to residents who rated it as ‘acceptable’ with a mean SUS score of 73.4 [23]. This difference was attributed to navigation challenges, as more experienced clinicians struggled with the interface despite their familiarity with the EHR system. Similar studies report that clinicians used 346 mouse clicks, including 200 left clicks, and visited 43 screens during a single documentation task [23, 26]. The clinicians toggled between two screens three times within 60 s because information was not easily accessible in one place, forcing clinicians to jump back and forth between tabs to gather data, which hindered efficiency [23, 26]. Sood et al. (2024) reported that social determinants of health (SDHs) and environmental determinants of health (EDHs) were perceived as difficult to chart, with low ease‐of‐charting scores due to the challenges clinicians faced when navigating and entering information on these determinants within the EHR [34].

This navigation challenges across various studies have been attributed to poorly designed EHRs functionalities such large free‐text fields and open‐ended documentation areas, which led to variability in the information recorded, but also increased the likelihood of errors [45]. For instance, templates used to document visits were not user‐friendly due to cumbersome design and excessive clicks, which hindered efficient documentation and led to provider frustration [32, 46]. A significant portion of templates contained placeholders for manual text entry, which increases the likelihood of documentation errors and places a higher burden on providers to input information accurately and completely [41]. The high number of irrelevant alerts for drug interactions, not prioritized by severity, along with poorly designed templates, led to physicians overlooking crucial information and making documentation errors [32, 36]. Hence, there is a need to reduce unnecessary alerts in EHR that appear during documentation and to standardize note templates to facilitate documentation, particularly for less experienced providers and trainees [32].

### 3.6 Fragmented Information Hinders Retrieval, Increasing Documentation Time and Errors

Clinicians expressed frustration with information scattered across multiple EHRs, stating that this forces them to spend extra time locating and synthesizing information across various sources, which contributes to the documentation burden [31, 35, 44, 47]. For example, laboratory results, radiology reports, and medication lists stored in separate systems outside the main EHR complicated both the retrieval of patient information and the documentation process, thereby increasing the risk of overlooking critical data [39]. Difficulty in extracting meaningful data from a large number of notes, sorting and locating relevant documents, and navigating confusing text resulted in clinicians resorting to paper lists, which they kept in their drawers [31]. Furthermore, notes are stored by title and arranged chronologically without regard to their relevance to the problem at hand, so a provider might have to sort through 30 physical therapy notes to get to the last primary care visit; making it difficult for readers to discern the full thoughts and intent of other clinicians [31]. This has led clinicians to resort to verbal communication (e.g., nurses relaying the most recent vital signs to doctors) due to the fragmented nature of EHR data entry and retrieval [30].

Lybarger et al. (2018) also reported that, due to the asynchronous nature of automatic speech recognition (ASR), the documentation process became fragmented, with some data being added post‐visit rather than in real‐time [43]. This lack of seamless integration contributed to an increase in total documentation time, requiring clinicians to revisit and edit notes multiple times [43]. A similar study reported that EHR screens were accessed multiple times for some patients, with clinicians needing to revisit previously viewed screens due to relevant information being scattered across different locations [42]. As a result of the EHR's fragmented structure, the time required for documentation increased, with clinicians spending an average of 6 min and 27 s pre‐rounding each patients [22].

## 4 Discussion

Our review fills a critical gap by mapping existing knowledge about EHR‐related documentation burden and identifying usability and design issues that contribute to this burden. We identified four interlocking challenges: frequent task switching driven by navigational dead ends; fragmentation of clinically essential data across multiple screens; information overload due to copy‐paste and template auto population; and cognitive strain caused by misalignment between interface logic and clinical reasoning. These problems form a causal chain, fragmented interfaces prompt task switching, which inflates cognitive load, while redundant data further compounds mental effort, ultimately extending documentation time and increasing error risk.

Researchers continue to debate how strongly rapid EHR‐driven task switching undermines clinical efficiency and outcomes. Log analyses indicate that frequent within‐chart switches lead to cognitive overload, with clinicians repeatedly reopening the same order or documentation module just minutes after exiting, thereby prolonging disposition times [51]. Supporting this, a multimethod eye‐tracking study of ward nurses revealed that although multitasking accounted for only 39% of their duty time, multitasked EHR interactions tended to be shorter yet more frequent, fragmenting nurses' attention across competing demands [52]. Another study using the TimeCaT measurement instrument reported that medical‐surgical nurses make fewer switches on average than emergency department physicians, indicating that task density, patient turnover, and staffing ratios significantly influence switching rates [53]. Chaiyachati et al. (2019) observed internal medicine interns for 2000 h and found that 43% of their 24‐h shifts were spent using the EHR, typically in sessions lasting less than 90 s before switching to another task. These micro‐interruptions consume working memory and may contribute to cognitive overload [54, 55]. While consolidating EHR interfaces into integrated ‘dashboard’ views may reduce unnecessary switching, such changes alone risk simply shifting documentation tasks to scribes or assistants without truly reducing the burden [56, 57]. Future research should combine interface redesign with role reallocation to identify the most effective strategies for minimizing task‐switching.

Studies across various clinical settings show that when the EHR task sequence misaligns with clinical workflow, clinicians create workarounds, such as using paper, spreadsheets, or texting, to keep pace, thereby contributing to burnout [58, 59, 60]. For example, a longitudinal ethnography conducted in two Dutch hospitals mapped cycles in which exporting task lists to Excel and retyping results back into the chart increased documentation time and copy errors [61]. A study conducted in the U.S. Veterans Health Administration reported that 54 clinicians continued to use hand‐crafted paper aids despite having access to the EHR. Investigators framed these artifacts not as mere noncompliance, but as workarounds that reveal unmet information needs [62]. Another study reported that 61% of workarounds arise from poorly designed EHR functionality rather than deficient training, leading to duplicative entry, external note‐taking, and copy‐forward behavior [63]. However, interventions that align documentation with clinical workflow, such as co‐locating documentation and ordering within a single workspace and automating data pulls from prior notes may reduce workarounds associated with the EHR documentation burden [64].

Our study findings show that EHR design flaws such as excessive navigation or clicks and inconsistent iconography contribute to documentation errors and increased documentation burden. Similarly, study conducted in acute and critical care settings highlighted that EHR usability issues, including data redundancy, poor workflow navigation, and cumbersome data entry, significantly contributed to documentation burden [65]. Another study, found that poor EHR usability, particularly in areas like data entry, workflow navigation, and user interface was associated with increased medication errors [18]. Interruptive pop‐up alerts also force clinicians to navigate through serial EHR screens during documentation, with 96% of such alerts being overridden [66]. It has been suggested that non‐interruptive alerts may reduce alert overrides by 23% compared to pop‐ups, without increasing user clicks.

When relevant data reside on different screens or even different EHR systems, clinicians spend additional time piecing them together and are more likely to miss or misinterpret key facts; thereby contributing to workflow fragmentation. A similar study conducted in an ICU documented a median of 26.5 separate screens per chart‐review session, with frequent back‐and‐forth when switching between notes, labs and imaging [67]. In a pre‐rounding study, resident doctors required an average of 6 min and 27 s and 28 separate screens to assemble a single patient “snapshot,” largely because labs, medications, and notes could not be viewed together [22]. Another study argues that this scattering of information across screens is driven less by screen layout and more by upstream interoperability gaps that isolate laboratory, imaging, and pharmacy data in separate silos [68]. Addressing fragmentation is therefore central to any strategy aimed at alleviating documentation burden and improving patient safety.

### 4.1 Implication for Future Research

Although the evidence base around EHR‐related documentation burden is growing, several important questions remain. There are relatively few longitudinal studies examining how interface‐related demands such as high click loads or frequent task switching affect clinical outcomes, including error rates, clinician burnout, and staff turnover. The balance between standardized templates and user‐driven customization also remains poorly understood, with little empirical evidence on what configurations work best across different medical specialties. While early evaluations of AI‐based documentation tools, such as ambient scribing, have shown encouraging results, most studies lack randomized designs, long‐term follow‐up, or cost‐effectiveness analysis. Additionally, audit log data offer a rich source of behavioral telemetry, but the absence of standardized usability or cognitive load metrics limits the ability to compare findings across studies. Addressing these gaps will require mixed‐method research designs that integrate ethnographic observation, human factors analysis, and rigorous quantitative metrics.

### 4.2 Study Strengths and Limitations

This is the first scoping review that has sought to establish what is known about usability issues in EHR that contribute to documentation burden and clinical workflow disruption, to the best of our knowledge. Several disparate reviews and evidence syntheses have been conducted in recent times exploring different facets of EHR, but without a specific focus on usability in relation to documentation burden and clinical workflow disruption. Contemporary scoping reviews have focused on different aspects of EHR such as their implementation [69], impact on clinician burnout [70, 71], effect on information practices in mental health settings [72], as well as documentation burden measurement [73] and mitigation [74]. While these reviews have each reported their own distinct results that can be used to inform policy and practice, none of them has specifically focused on how usability impact on documentation and workflow disruption [69, 70, 71, 72, 73, 74]. Therefore, this scoping review makes a unique original contribution which builds on these reviews, by generating novel results that can be used to inform the design and effective implementation of EHR so that it brings about the best possible outcomes for patients as well as staff. We begin the discussion with a summary of our key results which are then duly critically analyzed in relation to pertinent wider literature.

Usability issues are complex and often shaped by a range of socio‐technical factors, including the physical work environment, resistance to change, fragmentation of care, and diverse user needs. These factors may not be fully captured in our study, particularly in terms of their interplay with the documentation burden. Additionally, our findings are largely based on studies conducted in high‐resource settings, such as the U.S., and may not fully capture usability challenges in low‐resource healthcare systems or in diverse clinical environments with varying EHR implementations. Another limitation is our reliance on three databases and limited hand‐searching, which may have led to the omission of relevant studies published in other databases or in the grey literature.

## 5 Conclusion

This study highlights the significant impact of EHR usability issues on documentation burden and clinical workflow disruptions. Addressing these challenges requires embedding human factors methods into the EHR design and implementation cycle. By doing so, developers and healthcare organizations can better align EHR systems with clinical workflows, reduce unnecessary documentation burdens, and enhance patient safety. This holistic approach must extend beyond merely adding new features and functions; it should prioritize how clinicians interact with EHR systems to ensure they are safer, more efficient, and more supportive of clinicians' decision‐making and communication needs.

## Author Contributions

Conceptualization: The study was conceptualized by OO. Data Curation: Data collection and analysis were carried out by OO, SA, and RS. Methodology: The methodology was developed by OO. Writing – Original Draft: The initial draft of the article was written by OO. Writing – Review and Editing: The article was critically reviewed and edited by RS, HI, OO, and SA.

## Conflicts of Interest

The authors declare no conflicts of interest.

## Data Availability

The authors have nothing to report.

## Appendix A Study characteristics

**Table:**

Authors	Objectives	Methodology	Study setting	Study location	Concise summary of findings
Jawad Alami, et al. 2022 [22]	Identify usability challenges associated with the design of Electronic Health Records (EHRs) during pre‐rounding on pediatric inpatients.	Thirty residents think‐aloud protocol. Surveys, video recordings, and comments were analyzed to identify EHR usability issues.	Thirty pediatric residents at University of Virginia	Charlottesville, VA (USA)	Prerounding was inefficient and frustrating due to EHR design limitations. Recommendations included improving data accessibility, creating a checklist, standardizing notes, and automating calculations.
Rubina F. Rizvi et al. 2017 [23]	Evaluate usability of an EHR's clinical notes interface for attending and resident physicians to improve EHR usability using user‐centered design (UCD) principles.	Scenario‐based usability testing on clinical documentation tasks using two test patient charts in an Epic system environment.	Fairview Health Services, University of Minnesota Medical Center	Minneapolis, MN (USA)	Residents perceived the EHR system as more usable than attendings. Efficiency improved with repeated tasks, though differences in perceived workload for note types indicated training needs. The study highlights variability in EHR usability based on user characteristics.
Yalini Senathirajah, et al. 2020 [26]	Define and analyze sources of fragmentation in EHR user interfaces to optimize or redesign systems for improved usability.	Mixed‐methods study using sunburst visualizations to capture EHR navigation and time belt visualizations to assess task fragmentation.	Various clinical settings in the United States and Canada	Multi‐site study	Visualization techniques revealed high fragmentation in conventional EHRs, suggesting inefficiencies in task navigation and usability. Recommended user‐centered redesigns to reduce cognitive load and improve workflow.
Charlene R. Weir et al. 2017 [31]	Identify challenges in electronic documentation systems and assess provider experiences and perceptions of the Veterans Health Administration (VHA) EHR.	Interviews with 88 providers at 10 VHA primary care sites	Veterans' Health Administration (VHA) primary care clinics	Multiple VA sites in the United States	Providers reported major challenges in navigating electronic documentation due to information overload, hidden data, and mistrust in EHR accuracy, highlighting issues with copy‐pasting and templated data that may undermine patient care quality.
Natasha Sood et al. 2024 [34]	Examine the influence of provider perceptions of EHR usability and clinical importance of Social and Environmental Determinants of Health (SEDH) on charting practices.	Cross‐sectional survey study assessing self‐reported practices related to SEDH documentation among 201 physicians	Penn State Health Hershey Medical Center	Hershey, PA (USA)	SEDH documentation frequency was influenced by perceived clinical importance and EHR usability. However, there were discrepancies, with social determinants of health (SDH) charted more consistently than environmental determinants (EDH), revealing opportunities for EHR design and training interventions.
Daniel R. Murphy et al. 2019 [47]	Identify barriers and facilitators impacting the reliability of the EHR‐facilitated total testing process (TTP).	Exploratory study using expert interviews at three large EHR‐enabled health care organizations	Three large healthcare organizations in Texas	Texas, USA	Barriers related to EHR usability and communication between clinicians and diagnostic services were noted, increasing TTP vulnerability. Facilitators included EHR features and improved workflows. Recommendations were made for focusing on interface improvements and EHR usability to increase TTP reliability.
David R. Kaufman et al. 2015 [42]	Analyze sequential patterns in clinician EHR screen transitions to better understand workflow inefficiencies.	Sequential data analysis using process mining tools on screen transitions in EHR during pre‐rounds in Mayo Clinic's Colon and Rectal Surgery unit.	Mayo Clinic, Colon and Rectal Surgery Division	Rochester, MN (USA)	Identified common screen transition patterns that varied in complexity and efficiency. Findings suggest potential workflow inefficiencies and indicate that specific EHR redesigns could better support clinicians' sequential information needs during patient rounds.
Jean E. Stevenson et al. 2016 [30]	Identify and describe workarounds in EHR documentation of vital signs in response to usability issues in a hospital setting.	Qualitative study using observations and interviews at a 372‐bed hospital in Sweden.	372‐bed district hospital, covering cardiology, infection ward, and emergency department	Kalmar, Sweden	Nurses and doctors developed workarounds like paper notes and charts to efficiently document and retrieve vital signs, addressing EHR usability issues. These practices facilitated workflow but created potential for delays and inconsistencies in patient records.
Karen Dunn et al. 2021 [48]	Assess the impact of EHR usability and workload for providers and nursing staff following a transition to a new EHR over an extended period.	Heuristic evaluation identified EHR design flaws influencing usability and workload	Ambulatory urgent care centers within one healthcare system	Small Midwestern metropolitan area, USA	Significant increases in perceived workload were observed post‐implementation, persisting for 2.5 years. Providers experienced higher frustration and temporal demand than nursing staff, linked to design flaws identified in heuristic analysis, highlighting the need for EHR redesigns to alleviate clinician burden.
Brian Lefchak et al. 2023 [35]	Characterize usability perceptions among ambulatory clinical staff using EpicCare and Allscripts EHR systems, analyzing differences by provider role.	Cross‐sectional survey study with a customized 19‐question survey on usability constructs	NewYork‐Presbyterian Hospital, Weill Cornell Medical Cente	New York, NY, USA	Usability perceptions varied significantly by role and EHR system, with higher usability scores for EpicCare. Ordering providers (OPs) perceived lower usability than non‐OPs, particularly in user control and cognitive support, indicating potential areas for EHR interface improvement to reduce cognitive burden and improve satisfaction.
A. Jay Holmgren et al. 2024 [36]	Measure EHR usability, satisfaction, and burnout among family physicians, evaluating associations with efficiency strategies.	Cross‐sectional survey conducted among family physicians undergoing recertification in 2022.	Family medicine practices across the United States	Multi‐site, USA	Variability in EHR satisfaction was noted, with only a quarter of respondents very satisfied. Efficiency strategies improved satisfaction only among physicians with highly usable EHRs. High satisfaction correlated with reduced burnout, suggesting usability and efficiency as key to alleviating physician EHR burden.
Tom Ebbers et al. 2024 [49]	Evaluate the impact of a multidisciplinary, EHR‐embedded care pathway to improve structured data recording and reduce EHR burden in head and neck oncology care.	Before‐and‐after study involving video analysis of consultations in a tertiary oncology center, assessing usability metrics	Tertiary oncology center using HiX EHR	Nijmegen, Netherlands	Implementation of an EHR‐embedded care pathway led to a 27% reduction in EHR time per initial oncology consultation, improved structured data capture, and enhanced usability perceptions among providers. Time savings were primarily attributed to reduced time on order entries and improved input efficiency.
Katelyn N. Hettinger et al. 2023 [24]	Conduct a formative usability evaluation of an HIE interface prototype developed for community pharmacists and pharmacy technicians.	Scenario‐based usability evaluation using think‐aloud protocols	Community pharmacies within Indiana's PioneerRx network	Indiana, USA	The HIE‐Pioneer mock‐up showed generally positive usability, with 23 usability issues identified. High‐impact issues included excessive clicks to access HIE data and ambiguous tool names. The study suggests that integrating HIE data within pharmacy systems can enhance workflow and supports the need for an optimized interface.
Tom de Hoop, et al. 2020 [39]	Evaluate physician time expenditure and EHR limitations in a German university medical center and propose a tool to capture limitations at the point of care.	Time‐and‐motion study observing 19 physicians across six department and conducting semi‐structured interviews.	University Medical Center, Leipzig University	Leipzig, Germany	Physicians spent 37% of time on EHR tasks, including extensive time searching, reading, and writing. Major limitations included fragmented information systems, high documentation burden, and challenges in information exchange. Suggested development of a tool for real‐time issue reporting to address limitations at the point of care
Jessica Schwartz et al. 2020 [40]	Develop an interprofessional taxonomy to assess EHR documentation burden and measure clinician workflow among nurses and physicians in a large medical center.	Time‐motion study using an interprofessional task taxonomy with five observers to track time and tasks in EHR.	Large academic medical center	New York, NY, USA	Developed and validated a reliable taxonomy to track both nursing and physician workflow tasks related to EHR use. Findings indicated substantial documentation time, supporting the taxonomy's use for identifying workflow inefficiencies and shared documentation burden across clinical roles.
Tania Tajirian et al. 2020 [33]	Investigate the impact of EHR use on physician burnout and assess EHR‐related burnout contributors among mental health physicians and learners.	Cross‐sectional survey with 208 participants at a mental health hospital,	Centre for Addiction and Mental Health	Toronto, Canada	Findings showed a strong link between EHR dissatisfaction and physician burnout, with 69% attributing EHR to burnout symptoms. Usage logs revealed overestimated EHR time by physicians, highlighting discrepancy in perceived versus actual time. Recommendations included refining EHR to ease administrative burdens and enhancing user training and support.
Adam Rule et al. 2022 [41]	Describe how primary providers and other members of the care team use note templates to document outpatient visits and identify documentation patterns.	Retrospective cross‐sectional study using EHR metadata from Oregon Health & Science University	Oregon Health & Science University	Portland, OR (USA)	Found high fragmentation in template use, with 83% of templates used only by individual users. Most visits (89%) used at least one template, with variability across specialties. Recommended exploring standardization while supporting customization to reduce EHR burden.
Shivang U. Danak et al. 2019 [27]	Assess the impact of medical scribes on patient‐physician communication in primary care encounters.	Convergent mixed methods study, Patient surveys, video‐recorded interactions, and physician interviews examined	Primary care, large academic medical center	Midwestern USA	Patient satisfaction with communication was high in both scribed and non‐scribed groups. Scribes had minimal effect on encounters, but helped physicians focus more on patients. Physicians found scribes valuable for reducing documentation burden, with minimal impact on patient experience.
Cecilia Anne Kennedy Page et al. 2014 [45]	The study aimed to enhance the usability of Electronic Medical Records (EMRs) in nursing practice to improve efficiency, effectiveness, and satisfaction with the documentation process.	2‐year phased redesign (4 phases) across patient populations; iterative design, user feedback	University of Kentucky Healthcare	Lexington, Kentucky, USA	The usability‐focused EMR redesign reduced documentation time by 45.2%, improved patient outcomes (e.g., 30% fewer infections, 43.8% fewer pressure ulcers), and increased nurse satisfaction by up to 22.6%. This approach shows that EMR usability improvements can enhance efficiency, patient safety, and user satisfaction.
Kana Kodama et al, 2023 [44]	Develop and assess a self‐reported EMR‐connected questionnaire to reduce nurses' documentation time	EMR‐connected questionnaire system allowing patient self‐reporting, implemented in a hospital setting, and usage tracked to compare documentation time between patients who used the system and those who did not	Osaka University Graduate School of Medicine and affiliated hospitals	Suita, Japan	The system reduced documentation time for nurses, particularly in internal medicine and dependent ADL patients. Additionally, 88% of patients found the questionnaire easy to use, with 85% willing to use it again. The system proved efficient in both usability and time reduction, suggesting benefits for broader adoption in clinical settings.
Kevin J. Lybarger et al. 2018 [43]	Analyze physician editing of clinical notes created using asynchronous automatic speech recognition (ASR)	Study of 649 ASR‐generated notes edited by 9 physicians, focusing on edit types and frequency	University of Washington Medical Centers, internal medicine inpatient services	Seattle, Washington, USA	Physicians added 40% of final content during editing: 6% short edits for error correction, 34% extended edits to add clinical data. Majority of changes were in the “Assessment & Plan” section. Findings suggest ASR edits reduce time but would benefit from automation to improve efficiency and accuracy.
Mary Regina Lindsay et al. 2022 [38]	To improve efficiency, reduce redundancy, and streamline nursing documentation in EHR	Quality improvement pre‐/post‐intervention design, with timestamp and video recording analysis	Duke University Health System, including 3 hospitals and over 150 ambulatory sites	Durham, North Carolina, USA	Post‐intervention, documentation time in the EHR decreased by 18.5% overall, with 10‐12% reduction in flowsheet time. Steps to complete documentation decreased by up to 97%, saving nurses 1.5 to 6.5 min per reassessment. Improvements contributed to reduced workload and enhanced usability, supporting more direct patient care activities.
Francesca M. Nicosia et al. 2019 [32]	Explore clinician perspectives on barriers and facilitators to measuring functional status in primary care for older adults	Qualitative study using semi‐structured interviews with 24 clinicians across 6 VA medical centers	Primary care and geriatrics clinics at VA Medical Centers	United States	Clinicians identified key barriers to functional status measurement: time constraints, lack of standardized processes, and limited integration of data into clinical workflows. Facilitators included structured templates, interdisciplinary approaches, and improved usability.
Paulina S. Sockolow et al. 2020 [28]	Evaluate the impact of EHRs on nurse clinical processes and satisfaction at two community health sites	Mixed methods design, including quantitative data on documentation time and nurse satisfaction surveys; qualitative observations and interviews	PACE (Program of All‐inclusive Care for the Elderly) and a home care agency	Philadelphia, Pennsylvania, USA	EHRs improved documentation timeliness, but nurses faced usability issues and workflow mismatches, impacting efficiency and satisfaction. Barriers included complex navigation and lack of interoperability. Facilitators included documentation prompts and the ability to access other clinicians' notes.
Gina M. Berg et al. 2020 [46].	Identify usability issues in EHR systems as perceived by resident physicians to understand barriers in workflow and potential impact on burnout	Survey and focus group with resident physicians to identify usability principal violations and document their impact on workflow and satisfaction	Multiple residency programs at a medical school in the Midwest	Wichita, Kansas, USA	Residents identified usability issues, including increased documentation time, confusion, and alert fatigue, particularly in Meditech and CPRS systems. Key issues were excessive pop‐up warnings, lack of consistency in terminology, and ineffective navigation, all leading to disruptions in workflow and potential patient safety concerns.
